# Biostatistics as a Tool for Medical Research: What are we Doing Wrong?

**DOI:** 10.31138/mjr.30.4.196

**Published:** 2019-12-31

**Authors:** Paraskevi Tsiamalou, Alexandros Brotis

**Affiliations:** 1 Department of Rheumatology and Clinical Immunology, General University Hospital of Larissa, Larissa, Greece,; 2 Department of Neurosurgery, General University Hospital of Larissa, Larissa, Greece

**Keywords:** biostatistics, research, statistics, credibility

## Abstract

As the data resulting from modern clinical research are astonishingly complex, statistics constitute an integral component of the research project, from the study conception and design to the reporting of the results. At the same time, the Medical Literature is not immune to statistical pitfalls. In the following lines we identify eleven common statistical mistakes, elucidate their effects on the study credibility, and provide tips and tricks to avoid them.

## INTRODUCTION

Modern clinical research requires the extensive use of statistics. This is understandable, as on the one hand, authors are sometimes characterized by an overzealous interest in achieving a large volume of publications, and on the other hand, Journals have set high standards for manuscript acceptance.^1^ However, quantity does not go hand-in-hand with quality, and the underlying statistical analyses have frequently been considered as suboptimal.^2^ This problem is persistent, serious, and unknown to the new researcher, despite the fact that most errors concern basic statistical concepts and can be easily avoided with the proper training.^1–3^ Only a few authors have a deep understanding of the various study designs and the underlying statistical concepts. A substantial part of the problem is accounted for by the broad availability of statistical software and its extensive use by the uninitiated. Of note, statistical mistakes in clinical research are un-ethical, costly in terms of time and resources, and harmful to humanity and science.^2^ In this review, we focused on eleven common statistical pitfalls met in the Medical Literature, and provided tips and tricks on how to avoid them. For practical reasons, the pitfalls were categorized into five groups: mistakes in the study design, data collection, statistical analysis, interpretation of the results, and reporting of the results. Finally, we enriched our manuscript with a few examples from the Medical Literature related to the field of Rheumatology.

## STUDY DESIGN

### Inappropriate study design

Frequently, the aim of study is to test a null hypothesis (H_0_) against the alternative (H_1_) using a particular set of data under the proper study design. Flaws during the study design result in fundamental errors, which are difficult to correct during the statistical analysis process. Selection of the appropriate target and control groups constitutes a pivotal step in the study design process. Equally important decisions are related to the choice of the optimal randomization, blinding, and matching methods. It is worth mentioning that clinical efficacy (“*can it work?”*) is demonstrated under “optimal circumstances” in randomized controlled trials, while cohort studies are reserved for effectiveness (*“does it work?*”). Similarly, clinical efficiency (*“is it worth it?*”) is assessed using economic evaluation studies, including cost-benefit, cost-effectiveness, and cost-utility analyses. Kingsley et al. conducted a 6-month, double-blind, parallel-group randomized controlled trial to evaluate the efficacy of methotrexate (MTX) in patients with psoriatic arthritis (PsA).^4^ Patients with active PsA were randomized to receive MTX or matched placebo, with an allocation ratio of 1:1. Patients were randomly allocated using random number tables. The researchers and the trial coordinator were unaware of the allocation sequence. Treatment assignments were in a locked cabinet in the coordinating center pharmacy for emergency access.

### Underpowered studies without an a priori sample size estimation

An inherent component of the study design process is the estimation of the study sample to assure an adequate power and detect statistical significance. The required sample size depends on the acceptable level of type II error, the difference or effect of interest, and the estimated variability of the outcome variable. Ideally, the sample size is calculated to obtain estimates of desired precision, detect an effect if it really exists, and should be large enough to compensate for dropouts. Small-sized studies may not have enough power to reach safe statistical conclusions, whereas unjustifiably large samples lead to unnecessary waste of resources. Hewlett et al. performed a randomized controlled trial to study if a group course delivered by rheumatology teams using cognitive-behavioural approaches (CBT), plus usual care, reduced the rheumatoid arthritis fatigue impact more than usual care alone.^5^ The authors estimated that 73 patients/arm would detect 1.46 units difference in fatigue impact (90% power and a two-sided significance of 0.05). They finally aimed to recruit 150/arm after considering for potential clustering effects from groups/tutors and allowing for 2-year attrition (50%).

## DATA COLLECTION

### Missing values

Missing values constitute an essential problem in the data collection process, as they reduce the total power of the study and may introduce bias. The severity of the problem depends on the nature and the magnitude of the ‘missingness’. In general, there are three types of missing data according to the mode of occurrence: missing completely at random (MCAR), missing at random (MAR), and missing not at random (MNAR).^6^ In the case of MCAR, the probability that the data are missing is not related to either the specific value, which is supposed to be obtained, or the set of observed responses. MAR occurs when the probability that the responses are missing depends on the set of observed responses, but is not related to the specific missing values. If the missing data are not MCAR or MAR, then they fall into the category of MNAR. There is no optimal method to deal with missing data. By far the most common practice is to perform an analysis based on the complete cases. Alternatively, some form of missing data imputation is recommended.^7^ Mean imputation, regression imputation, and last observation carried forward, just to name a few, have been sporadically used in the Medical Literature. In the aforementioned study by Kingsley et al., the authors assumed that unobserved measurements were missing at random, and imputed missing data by multiple imputation using chained equations with 20 cycles to create an equal number of imputed data sets.^4^ The latter were separately analysed and the results were pooled using Rubin’s rules.^6^

### Categorization of continuous variables

Categorization of continuous variables is a common practice in clinical research. It simplifies the statistical analysis and facilitates interpretation and reporting of results. However, it leads to several serious statistical problems, including the loss of study precision, blooming of the type I error, and concealment of non-linearity between the dependent and independent variables. Categorization is justified when the data are markedly skewed, the variables show a nonlinear relation to another, and when the values are “guesstimates” or imputations of missing data. The use of “optimal” cut-points that results in minimal p-values is strongly discouraged. If a cut-point is to be used, then it is preferable to use clinically important thresholds. The body mass index (BMI) is an example of a continuous variable that is frequently analysed as a categorical variable.^8^ Accordingly, Feng et al. reviewed the role of BMI on the risk for rheumatoid arthritis (RA) by conducting a meta-analysis of thirteen observational studies.^9^ The authors reported that the relative risk for RA was 1.21 (95% CI: 1.02–1.44) and 1.05 (95% CI: 0.97–1.13) for obese and overweight patients, respectively. In addition, they reported that the risk of RA increased by 13% (1.13; 95% CI: 1.01–1.26) for every 5kg/m^2^ increase in BMI.

## STATISTICAL ANALYSIS

### Violation of statistical assumptions

Most parametric tests are valid given that certain assumptions are fulfilled, including the assumption of normality, independence, linearity, and equality of variance or homoscedasticity. Shapiro-Wilk and Smirnoff-Kolmogorov tests can be used to test for the assumption of normality distribution.^10^ The linearity assumption can be assessed by inspection of the “residuals over fitted” plot. Equality of variance may be evaluated by the F-test to compare the variances of two samples, and the Bartlett’s test or Levene’s test to compare the variances of multiple samples.^11^ Non-parametric tests, characterized by a low statistical power in small samples, are used when the statistical assumptions are not fulfilled. Violation of these assumptions leads to erroneous results and conclusions. De Morais-Barbosa studied the effect of foot orthoses on balance, foot pain and disability in ninety-four elderly women with osteoporosis through a randomized clinical trial.^12^ Patients were randomly assigned to an intervention group with foot orthoses or to a control group without orthoses. The researchers used Berg Balance Scale, the Timed Up and Go test, the Manchester Foot Pain and Disability Index and a numeric pain scale at baseline and after 4 weeks. Because of the absence of normal distribution in their measurements, the authors preferred to analyse the variables after converting them into ranks.

### Failure to detect dependencies

The statistical unit, defined as the entity on which information is received, constitutes a frequently under-reported parameter in the Medical Literature. Individual patients form the most commonly used statistical units. Failure to recognize the statistical unit frequently leads to concealment of dependencies, and manipulation of dependent variables as independent, at the cost of valuable information. Data from repeated measurements and matched groups are examples of dependent data that require special handling. Marouen et al. reported that sodium excretion was higher in 24 patients with RA than in an equal number of matched controls.^13^ Unfortunately, the authors did not appreciate the dependency of the two groups, and used unpaired statistical analysis, obliviating the benefit of matching.

### Multiple comparisons

Every statistical test carries a nonzero probability of incorrectly detecting significance by chance (type I error). Performing multiple comparisons increases this potential error and should be avoided. A number of specialized tests and adjustments are available, differing in the terms of how they control the overall type I error rate. Dunnett’s test is used to compare each of several experimental conditions with a control.^14^ Tukey or Duncan tests are used to compare all pairs of experimental conditions, depending on the number of desired comparisons and the sample sizes.^14^ Bonferroni adjustment is another valid approach, according to which the significance level is reset at 0.05/N, where N represents the number of comparisons of interest.^14^ Alternatively, researchers are advised to recognize the risk for false positive findings at the limitations section. In the study by de-Morais-Barbosa, the authors used repeated measures of analysis of variance followed by Tukey’s test for multiple comparisons and the contrast profile test to compare the longitudinal measures.^12^

## INTERPRETATION OF THE RESULTS

### Incorrect interpretations of p-value

P-values have been abused in multiple ways.^15^ To start with, the statistical significance estimated by the p-value is not equivalent to clinical relevance and importance. P-values focus solely on statistical hypothesis testing, fail to convey important quantitative information, and do not provide evidence of directionality (one-tailed or two tailed). Thus, a very small p-value does not necessarily represent a strong difference (or association) between two variables. Likewise, the absence of evidence (large p-value) is not synonymous with evidence of absence (no effect). This is particularly true when there is no estimation of the required sample size. Owlia et al. focused on the frequency of sacroiliitis among patients with low back pain.^16^and some have unspecific symptoms. The aim of this study was to determine the frequency of sacroiliitis causes among patients attending Shahid Sadoughi’s infectious disease and rheumatology clinics. METHODS In this study, we evaluated patients attending Shahid Sadoughi rheumatology and infectious diseases clinic in 2014. Patients who had positive findings in favor of sacroiliitis were evaluated by history, physical exam, laboratory tests, and imaging. The patients were divided into infectious, inflammatory non-infectious, and degenerative causes. The data were analyzed by IBM SPSS version 20 using the independent samples t-test, ANOVA, the chi-squared test, and the Fisher’s exact test. RESULTS We studied 136 patients. Among them 64 (47.1% The authors reported that the association of the gender and underlying aetiology (infectious, non-infectious, and degenerative) was not significant (p=0.147). However, this finding was pretty confusing, particularly when their contingency table included zero counts.

## REPORTING OF THE RESULTS

### Under-reporting of effect-size estimates

Clinical relevance and statistical significance are frequently depicted with the effect-size estimate along with its 95% confidence intervals, which are frequently under-reported. The results are sometimes evaluated for clinical relevance according to levels set by the researcher. The 95% confidence intervals denote that, when repeating the experiment, 95% of the samples will include the true value within their 95% CI. A wide confidence interval means that the sample size was too small, whereas a narrow interval is indicative of high precision. Consequently, many renowned journals have long discouraged the use of p-values and recommended effect size estimates and the 95% confidence intervals, instead.^15^ Wallace et al. studied the efficacy of baricitinib for systemic lupus erythematosus in a double-blind, randomized, placebo-controlled trial, recruiting from 78 centres in 11 countries.^17^ The authors revealed that in the 24^th^ week, the resolution of SLEDAI-2K arthritis or rash was achieved by 70 (67%) of 104 patients receiving baricitinib 4 mg (odds ratio [OR] vs placebo 1.8, 95% CI 1.0–3.3; p=0.041) and 61 (58%) of 105 patients receiving baricitinib 2 mg (OR 1.3, 0.7–2.3; p=0.39).

### Inappropriate use of SD and SEM

A continuous variable is traditionally described by a measure of central tendency (mean or median) and a measure of dispersion, such as standard deviation (SD). On the other hand, the standard error of the mean (SEM), an inferential statistic, is a measure of the mean’s precision. It is a frequent mistake to provide numbers without quoting what they stand for. Equally important, the standard deviation and the standard error of the mean are not synonymous and should not be used interchangeably.^18^ The SEM is the SD divided by the square root of the sample size. The choice of SEM over SD is ultimately dependent on what the researcher is trying to convey in their report. The SEM is calculated after dividing the SD by the square root of the sample size. Segal et al. studied the oxidative stress and fatigue in systemic lupus erythematosus through a case-control study.^19^ The authors reported that the mean age of the cases and the controls were 42.6 years (SEM: 2 years) and 41.7 years (SEM: 1.5 years), respectively. To our disappointment, the SEM did not provide any information on the age distribution around the mean values of the two groups.

### Poor tables and figures

Tables and figures are valuable tools in storing, analysing, and interpreting data. However, published articles should contain the minimal number of tables and figures to assist in proper communication of the study.^20,21^ Towards this aim, tables are frequently used for communicating precise numerical data, charts are optimized for presenting general patterns and comparisons, and maps are reserved for highlighting spatial relationships.^20^ Nevertheless, such a practice is not immune to potential errors.^21,22^ Frequently inexperienced researchers do not know how to match the proper graphic type with the available type of data, and end up using graphs at random, resulting in wrong impression of the true nature of the data. Finally, many graphical displays lack an adequate description of the legend, axes, and the underlying statistics.

## CONCLUSIONS

The majority of the statistical pitfalls in the medical literature are attributed to the poor statistical background of the authors. Emphasis should be given to the selection of the proper study design, estimation of the required study sample size before the enrolment of the first patient, avoiding categorization of continuous variables, and proper treatment of missing data. Statistical assumptions are mandatory for the selection of the appropriate statistical test and should be reported in the manuscript. A clear study aim is useful in avoiding multiple unnecessary comparisons.

It is high time to move from the p-value towards the effect-size estimate. Special care is required when reporting the distribution around the mean, and in displaying data in graphs and tables. Researchers are invited to update their knowledge with participation in statistical courses. In addition, each research group should include or co-operate with an expert Biostatistician from the study design until the reporting of the results. Finally, medical journals are encouraged to reject manuscripts with poor statistics. To achieve this, Editors should also include a Biostatistician in the review team, in order to recognize potential statistical pitfalls and recommend their proper correction.

## CONFLICT OF INTEREST

The authors declare no conflict of interest.

## References

[1] Altman DG. The scandal of poor medical research. BMJ 1994;308(6924):283–4. [cited 2019 Apr 25] [10.1136/bmj.308.6924.283] [PMID: ] [PMCID: ] 8124111 PMC2539276

[2] Ioannidis JPA. Why most published research findings are false. PLoS Med 2005;2(8):e124. [cited 2019 Apr 25] [10.1371/journal.pmed.0020124] [PMID: ] [PMCID: ] 16060722 PMC1182327

[3] Prescott RJ Civil I. Lies, damn lies and statistics: Errors and omission in papers submitted to INJURY 2010–2012. Injury 2013;44(1):6–11. [cited 2019 Apr 25] [10.1016/j.injury.2012.11.005] [PMID: ] 23182752

[4] Kingsley GH Kowalczyk A Taylor H Ibrahim F Packham JC McHugh NJ A randomized placebo-controlled trial of methotrexate in psoriatic arthritis. Rheumatology (Oxford) 2012;51(8):1368–77 [cited 2019 Apr 28] [10.1093/rheumatology/kes001] [PMID: ] [PMCID: ] 22344575 PMC3397466

[5] Hewlett S Almeida C Ambler N Blair PS Choy EH Dures E Reducing arthritis fatigue impact: two-year randomised controlled trial of cognitive behavioural approaches by rheumatology teams (RAFT). Ann Rheum Dis 2019;78(4):465–72. [cited 2019 Apr 28] [10.1136/annrheumdis-2018-214469] [PMID: ] [PMCID: ] 30793700 PMC6530078

[6] Rubin DB. Inference and Missing Data. Biometrika 1976;63(3):581 [cited 2019 Apr 28] [10.1093/biomet/63.3.581]

[7] Huque MH Carlin JB Simpson JA Lee KJ. A comparison of multiple imputation methods for missing data in longitudinal studies. BMC Med Res Methodol 2018;18(1):168. [cited 2019 Oct 18] [10.1186/s12874-018-0615-6] [PMID: ] [PMCID: ] 30541455 PMC6292063

[8] WHO | Obesity: preventing and managing the global epidemic . Report of a WHO Consultation (WHO Technical Report Series 894). [cited 2019 Apr 28] Available from: https://www.who.int/nutrition/publications/obesity/WHO_TRS_894/en/#.XMVfxthqGgM.mendeley 11234459

[9] Feng J Chen Q Yu F Wang Z Chen S Jin Z Body Mass Index and Risk of Rheumatoid Arthritis. Medicine (Baltimore) 2016;95(8):e2859. [cited 2019 Apr 28] [10.1097/MD.0000000000002859] [PMID: ] [PMCID: ] 26937917 PMC4779014

[10] Ghasemi A Zahediasl S. Normality tests for statistical analysis: a guide for non-statisticians. Int J Endocrinol Metab 2012;10(2):486–9. [cited 2019 Apr 25] [10.5812/ijem.3505] [PMID: ] [PMCID: ] 23843808 PMC3693611

[11] Wang Y Rodríguez de Gil P Chen Y-H Kromrey JD Kim ES Pham T Comparing the Performance of Approaches for Testing the Homogeneity of Variance Assumption in One-Factor ANOVA Models. Educ Psychol Meas 2017;77(2):305–29. [cited 2019 Apr 25] [10.1177/0013164416645162] [PMID: ] [PMCID: ] 29795915 PMC5965542

[12] de Morais Barbosa C Barros Bertolo M Marques Neto JF Bellini Coimbra I Davitt M de Paiva Magalhães E. The effect of foot orthoses on balance, foot pain and disability in elderly women with osteoporosis: a randomized clinical trial. Rheumatology 2013;52(3):515–22. [cited 2019 Apr 28] [10.1093/rheumatology/kes300] [PMID: ] 23192905

[13] Marouen S Cailar G du Audo R Lukas C Vial G Tournadre A Sodium excretion is higher in patients with rheumatoid arthritis than in matched controls. PLoS One 2017;12(10):e0186157. [cited 2019 Apr 28] [10.1371/journal.pone.0186157] [PMID: ] [PMCID: ] 29028829 PMC5640209

[14] Lee S Lee DK. What is the proper way to apply the multiple comparison test?. Korean J. Anesthesiol 2018;71(5):353–60. [cited 2019 Apr 25] [10.4097/kja.d.18.00242] [PMID: ] [PMCID: ] 30157585 PMC6193594

[15] Schober P Bossers SM Schwarte LA. Statistical Significance Versus Clinical Importance of Observed Effect Sizes. Anesth Analg 2018;126(3):1068–72.[cited 2019 Apr 25] [10.1213/ANE.0000000000002798] [PMID: ] [PMCID: ] 29337724 PMC5811238

[16] Owlia MB Danesh-Ardakani M. Frequency of sacroiliitis among patients with low back pain.. Electron physician 2016;8(3):2094–100. [cited 2019 Apr 28] [10.19082/2094] [PMID: ] [PMCID: ] 27123217 PMC4844474

[17] Wallace DJ Furie RA Tanaka Y Kalunian KC Mosca M Petri MA Baricitinib for systemic lupus erythematosus: a double-blind, randomised, placebo-controlled, phase 2 trial. Lancet 2018;392(10143):222–31. [cited 2019 Apr 28] [10.1016/S0140-6736(18)31363-1] [PMID: ] 30043749

[18] Sedgwick P. Standard deviation or the standard error of the mean. BMJ 2015 2 17;350:h831. [cited 2019 Apr 25] [10.1136/bmj.h831] [PMID: ] 25691433

[19] Segal B Thomas W Zhu X Diebes A McElvain G Baechler E Oxidative stress and fatigue in systemic lupus erythematosus. Lupus 2012;21(9):984–92. [cited 2019 Apr 28] [10.1177/0961203312444772] [PMID: ] 22508802

[20] Boers M. Designing effective graphs to get your message across. Ann Rheum Dis 2018;77:833–9. [cited 2019 Oct 18] [10.1136/annrheumdis-2018-213396] [PMID: ] 29748338

[21] Boers M. Graphics and statistics for cardiology: designing effective tables for presentation and publication. Heart 2018;104:192–200. [cited 2019 Oct 18] [10.1136/heart-jnl-2017-311581] [PMID: ] 29030423

[22] Franzblau LE Chung KC. Graphs, Tables, and Figures in Scientific Publications: The Good, the Bad, and How Not to Be the Latter. J Hand Surg Am 2012;37(3):591–6. [cited 2019 Apr 25] [10.1016/j.jhsa.2011.12.041] [PMID: ] 22305731

